# Novel Insights into *bla*_GES_ Mobilome Reveal Extensive Genetic Variation in Hospital Effluents

**DOI:** 10.1128/spectrum.02469-21

**Published:** 2022-07-26

**Authors:** Danieli Conte, Dany Mesa, Thomas Jové, Caetana Paes Zamparette, Thaís Cristine Marques Sincero, Jussara Kasuko Palmeiro, Libera Maria Dalla-Costa

**Affiliations:** a Faculdades Pequeno Príncipe (FPP), Curitiba, Paraná, Brazil; b Instituto de Pesquisa Pelé Pequeno Príncipe (IPPPP), Curitiba, Paraná, Brazil; c University of Limoges, INSERM, CHU Limoges, RESINFIT, Limoges, France; d Departamento de Análises Clínicas, Universidade Federal de Santa Catarina (ACL-UFSC), Florianópolis, Santa Catarina, Brazil; e Laboratório de Microbiologia Molecular Aplicada, Universidade Federal de Santa Catarina (UFSC), Florianópolis, Santa Catarina, Brazil; University of Pittsburgh School of Medicine

**Keywords:** ESBL, carbapenemase, integrons, MLST, multireplicons

## Abstract

Mobile genetic elements contribute to the emergence and spread of multidrug-resistant bacteria by enabling the horizontal transfer of acquired antibiotic resistance among different bacterial species and genera. This study characterizes the genetic backbone of *bla*_GES_ in *Aeromonas* spp. and Klebsiella spp. isolated from untreated hospital effluents. Plasmids ranging in size from 9 to 244 kb, sequenced using Illumina and Nanopore platforms, revealed representatives of plasmid incompatibility groups IncP6, IncQ1, IncL/M1, IncFII, and IncFII-FIA. Different GES enzymes (GES-1, GES-7, and GES-16) were located in novel class 1 integrons in *Aeromonas* spp. and GES-5 in previously reported class 1 integrons in Klebsiella spp. Furthermore, in Klebsiella quasipneumoniae, *bla*_GES-5_ was found in tandem as a coding sequence that disrupted the 3′ conserved segment (CS). In Klebsiella grimontii, *bla*_GES-5_ was observed in two different plasmids, and one of them carried multiple IncF replicons. Three Aeromonas caviae isolates presented *bla*_GES-1_, one Aeromonas veronii isolate presented *bla*_GES-7_, and another A. veronii isolate presented *bla*_GES-16_. Multilocus sequence typing (MLST) analysis revealed novel sequence types for *Aeromonas* and Klebsiella species. The current findings highlight the large genetic diversity of these species, emphasizing their great adaptability to the environment. The results also indicate a public health risk because these antimicrobial-resistant genes have the potential to reach wastewater treatment plants and larger water bodies. Considering that they are major interfaces between humans and the environment, they could spread throughout the community to clinical settings.

**IMPORTANCE** In the “One Health” approach, which encompasses human, animal, and environmental health, emerging issues of antimicrobial resistance are associated with hospital effluents that contain clinically relevant antibiotic-resistant bacteria along with a wide range of antibiotic concentrations, and lack regulatory status for mandatory prior and effective treatment. *bla*_GES_ genes have been reported in aquatic environments despite the low detection of these genes among clinical isolates within the studied hospitals. Carbapenemase enzymes, which are relatively unusual globally, such as GES type inserted into new integrons on plasmids, are worrisome. Notably, K. grimontii, a newly identified species, carried two plasmids with *bla*_GES-5_, and K. quasipneumoniae carried two copies of *bla*_GES-5_ at the same plasmid. These kinds of plasmids are primarily responsible for multidrug resistance among bacteria in both clinical and natural environments, and they harbor resistant genes against antibiotics of key importance in clinical therapy, possibly leading to a public health problem of large proportion.

## INTRODUCTION

Horizontal gene transfer through mobile genetic elements (MGEs), such as transposons (Tns), insertion sequences (ISs), and integrons (Ins), plays an important role in spreading antimicrobial resistance, a significant global threat to public health, animals, and the environment. The most clinically significant antimicrobial resistance genes (ARGs) are usually located on different MGEs that can move intracellularly or intercellularly. In hospital effluents, the presence of diverse selection pressures combined with a high concentration of pathogenic/commensal microbes creates favorable conditions for the transfer of ARGs and the proliferation of antibiotic-resistant bacteria (1).

Interactions between MGEs contribute to the rapid evolution of diverse multidrug-resistant pathogens in antimicrobial chemotherapy. ISs and Tns are discrete DNA segments that can carry resistance genes to genetic locations in the same or different DNA molecules within a single cell. Integrons harbored by plasmids, Tns, and other mobile structures are called “mobile integrons” (MIs) because MGEs promote their dissemination. Therefore, special attention has been given to MIs from natural environments to gather information on their ecology and diversity and understand their role in bacterial adaptation (2).

Integrons are genetic systems that allow bacteria to capture and express gene cassettes. They typically consist of an *intI* gene encoding an integrase that catalyzes the incorporation or excision of gene cassettes by site-specific recombination, a recombination site *attI*, and one or two promoters responsible for the expression of inserted gene cassettes. Several promoter variants that vary in strength have been identified, and integrons with weaker promoters often have higher excision activity of integrase (3).

GES-type β-lactamases are rarely encountered. To date, 51 variants have been described (http://bldb.eu/, last updated on 4 June 2022) (4), of which 17 have carbapenemase-hydrolyzing activity due to their amino acid substitutions (Gly170Asn or Gly170Ser) (5). *bla*_GES_ has been described as gene cassettes associated with class 1 integrons on plasmids with different types of replicons (6). Since the first description of Klebsiella pneumoniae from France in 2000 (7), several human outbreaks of GES-producing Gram-negative bacteria have been described worldwide (8–12). Some GES-type carbapenemases have been found in environmental matrices (13) and clinical isolates, most frequently associated with single occurrences (14).

Despite worldwide reports, GES enzymes are not among the most widespread carbapenemase families (14). In southern Brazil, epidemiological studies conducted by our group have shown a gradual increase in antimicrobial resistance in hospitalized patients in the last 20 years (15–17). However, GES enzymes were not found (18, 19). Although GES enzymes have already been reported in several countries, few studies have explored the genetic context of these ARGs, especially in Brazil. Therefore, we performed whole-genome sequencing (WGS) and analysis of GES-producing *Aeromonas* spp. and Klebsiella spp. from two hospital effluents to provide genetic information about resistance determinants. These enzymes may be associated with genetic elements that can provide mobility, facilitating their transfer to clinically relevant mobile vectors. Knowledge of the genetic contexts of *bla*_GES_ will enable a better understanding of the molecular mechanisms driving mobilization and the emergence of resistance genes in different microorganisms that, through hospital effluents, will reach wastewater treatment plants, the major interfaces between humans and the environment.

## RESULTS

### Antimicrobial resistance profile of GES type-producing *Aeromonas* spp. and Klebsiella spp.

In the seven isolates studied (*Aeromonas* spp., *n* = 5, and Klebsiella spp., *n* = 2), we observed a resistance to β-lactams and aminoglycosides, being that Aero28 and KPN47 were multidrug resistant. In addition, Aero28 was resistant to meropenem-vaborbactam and imipenem-relebactam, but not to ceftazidime-avibactam. All phenotypic tests to determine extended-spectrum β-lactamase (ESBL) and carbapenemases producers converged with the catalytic property of each GES variant. The Aeromonas veronii isolates were inhibited by EDTA due to an intrinsic metallo-β-lactamase (CphA) (Table 1).

**TABLE 1:** Susceptibility profile of *Aeromonas* spp. and Klebsiella spp. from hospital effluents

Strain, GES type	MIC (mg/L) of:^*a*^																ESBL^*e*^	Carbapenemases	
	AMI	CAZ	CAZ-AVI^*b*^	CFDC	CTX	CPM	CIP	CST	ERT	GEN	IMI	IMI-REL^*c*^	MER	MER-VAB^*d*^	POL	TIG		Class A	Class B
A. caviae Aero19, GES-1	4	8	0.5	2	4	1	0.25	0.25	0.03	0.5	0.12	0.12	0.03	0.12	1	0.5	+	−	−
A. caviae Aero21, GES-1	32	8	≤0.015	1	4	0.25	0.25	0.12	0.03	1	0.12	0.12	0.03	≤0.015	1	1	+	−	−
A. caviae Aero52, GES-1	8	16	0.25	2	4	64	0.5	0.5	0.03	1	0.12	0.12	0.5	2	1	0.5	+	−	−
A. veronii Aero22, GES-16	32	>64	≤0.015	1	>128	32	0.25	0.5	>64	32	>128	0.25	32	0.25	1	1	+	+	+
A. veronii Aero28, GES-7	>32	>64	≤0.015	0.06	64	16	4	0.5	>64	64	>128	>8	64	>32	2	1	+	−	+
K. quasipneumoniae KPN47, GES-5	>32	>64	1	0.5	>128	64	4	0.25	64	>128	32	2/4	32	2	2	8	+	+	−
K. grimontii KOX60, GES-5	8	>64	1	2	32	4	1	0.25	64	16	16	0.25	32	2	1	0.5	+	+	−
E. coli conjugate 21, GES-1	32	>16	ND	ND	4	<4	0.5	0.25	<0.25	2	<0.5	ND	<0.5	ND	1	<1	+	−	−
E. coli transformant 22, GES-16	2	0.5	0.25	0.015	0.12	0.06	≤0.06	0.25	0.03	0.5	0.25	0.12	0.06	0.03	1	≤0.06	+	−	−
E. coli transformant 28, GES-7	2	>32	2	0.12	4	0.25	≤0.06	0.12	0.015	2	0.25	0.25	0.03	0.03	1	≤0.06	+	−	−
E. coli transformant 60, GES-5	1	4	0.5	0.03	0.5	0.06	≤0.06	0.25	0.12	0.25	0.5	0.25	0.25	0.03	1	≤0.06	+	−	−
E. coli J53	1	0.5	0.12	0.12	≤0.06	≤0.03	≤0.06	0.12	≤0.015	0.25	≤0.12	0.12	≤0.015	≤0.015	2	≤0.06	−	−	−
E. coli TOP10	2	0.25	0.12	0.12	≤0.06	0.06	≤0.06	0.12	≤0.015	0.25	≤0.12	0.12	≤0.015	≤0.015	1	≤0.06	−	−	−

a AMI, amikacin; CAZ, ceftazidime; CAZ-AVI, ceftazidime-avibactam; CFDC, cefiderocol; CTX, cefotaxime; CPM, cefepime; CIP, ciprofloxacin; CST, colistin; ERT, ertapenem; GEN, gentamicin; IMI, imipenem; IMI-REL, imipenem-relebactam; MER, meropenem; MER-VAB, meropenem-vaborbactam; POL, polymyxin B; TIG, tigecycline; ND, not determined.

b The MIC of ceftazidime was measured with a fixed avibactam concentration of 4 mg/L.

c The MIC of imipenem was measured with a fixed relebactam concentration of 4 mg/L.

d The MICs of meropenem were measured with a fixed vaborbactam concentration of 8 mg/L except for K. grimontii KOX60, which was measured with a fixed vaborbactam concentration of 4 mg/L.

e (+), positive phenotypic test; (−), negative phenotypic test.

Furthermore, plasmid DNA from Aero22, Aero28, and KOX60 strains was successfully transferred by electroporation into Escherichia coli TOP10 cells, and conjugation experiments of *bla*_GES_-encoding plasmids were successfully performed only for Aeromonas caviae (Aero21); this plasmid harbored all the genes required to autonomously conjugate (GenBank accession no. CP068231). Additionally, repeated attempts to transfer resistance by conjugation or transformation were unsuccessful to Aero19, Aero52, and KPN47. GES-type ESBL transconjugants and transformants showed similar resistance profiles to cephalosporins as those of the donor strains. Moreover, GES-type carbapenemases transformants were susceptible to carbapenems in comparison to the donor strains (Table 1).

### Resistome and *bla*_GES_-encoding plasmid configurations.

Table 2 summarizes genome information of GES type-producing *Aeromonas* spp. and Klebsiella spp. The sequence analysis of plasmids of three A. caviae isolates carrying *bla*_GES-1_ showed that p1Aero19 and p1Aero52, nonmobilizable plasmids, presented tellurite resistance (*terB*). Conversely, the p1Aero21, a conjugative plasmid, harbored cobalt, zinc, and cobalt-zinc-cadmium resistance gene (*czcD*) and mercury operon (*mer*). All of these plasmids were nontypeable.

**TABLE 2:** Sequence type, genome characteristics, and *bla*_GES_-encoding plasmids of *Aeromonas* spp. and Klebsiella spp. from hospital effluents

Strain	Genome	Length (bp)	GC%	MLST	Inc group	Integron	Resistance genes^*a*^	GenBank accession no.
A. caviae Aero19	Chromosome	4,371,624	61.6	884			*bla*_MOX-5-like_, *bla*_OXA-504-like_	CP068232
	p1	110,559	58.9		Nontypeable	In2062	*bla*_GES-1_, *dfrA22*, *arr-6b*, *qacEΔ1*, *sul1*, *terB*	CP068233
A. caviae Aero21	Chromosome	5,123,400	60.6	885			*bla*_MOX-1-like_, *bla*_OXA-504-like_	CP068231
	p1	244,072	57.8		Nontypeable	In2029	*aacA7*, *bla*_GES-1_, *catB3*, *qacEΔ1*, *sul1*, *dfrA22*, *czcD*, *mer*	CP068231
A. caviae Aero52	Chromosome	4,386,545	61.6	884			*bla*_MOX-5-like_, *bla*_OXA-504-like_	CP066813
	p1	111,631	58.9		Nontypeable	In2062	*bla*_GES-1_, *dfrA22b*, *arr6b*, *qacEΔ1*, *sul1*, *terB*	CP066814
A. veronii Aero22	Chromosome	5,060,940	58.3	886			*aphA15*, *aadA1*, *bla*_OXA-1-like_, *bla*_OXA-12-like_, *ampS*, *cphA3*	JAEMTZ000000000
	p1	80,584	56.6		P6	In2059	*bla*_GES-16_, *qacEΔ1*, *sul1*, *dfrA21*, *aacA4'-8*, *bla*_OXA-17_, *aphA15*, *bla*_OXA-4_, *aadA1*, *bla*_TLA-1_, *tetC*, *aac(6′)-Ib-cr*, *mph*(E), *msr*(E)	JAEMTZ010000183.1
Aeromonas veronii Aero28	Chromosome	4,905,987	58.5	257			*bla*_OXA-12-like_, *ampS*, *cphA3*	JAEMUA000000000
	p2	9,413	54.1		Q1	In2061	*bla*_GES-7_, *aacA4'*, *qacEΔ1*, *sul1*, *aac(6′)-Ib-cr*	JAEMUA010000108
K. quasipneumoniae KPN47	Chromosome	5,696,094	57.5	5527			*bla*_OKP-B4,_ *bla*_SHV-5_, *rmtD*, *fosA6*, *oqxA*, *oqxB*, *ompK36*, *ompK37*, *gyrA*, *acrR*	CP066859
	p1	170,976	53.5		M1	In200	*qacEΔ1*, *sul1*, *bla*_GES-5_, *dfrA22*, *bla*_GES-1Δ2_, *aacA4'-3*, *aacA4'-3*, *bla*_TEM-1A_, *bla*_SHV-5_, *bla*_OXA-9,_ *rmtD1*, *aac(6′)-Ib-cr*	CP066860
K. grimontii KOX60	Chromosome	6,070,417	55.6	350			*bla*_OXY-6-1_, *oqxB*	CP067433
	p2	136,576	54.9		FII-FIA	In174	*bla*_GES-5_, *sμL*, *qacEΔ1*	CP067435
	p3	128,898	54.3		FII	In174	*bla*_GES-5,_ *sμL*, *qacEΔ1*	CP067436.1

a *czcD*, cobalt-zinc-cadmium resistance gene; *mer*, mercury operon.

BLASTn analysis showed low similarity (23% coverage and 98.4% identity) among two IncP6 plasmids, p1Aero22 from A. veronii and pKRA-GES-5 from a clinical K. pneumoniae strain (GenBank accession no. MN436715) (20). Only replication initiation and partitioning genes showed similarity. P1Aero22 presented other resistance genes, such as *bla*_TLA-1_ and *tetC*, in a composite transposon, IS*Kpn15*. The other important resistance genes were found in different integrons. The IncQ1 plasmid (p2Aero28) shares high similarity (100% coverage and 99.94% identity) with a plasmid from a clinical Klebsiella variicola isolate (GenBank accession no. CP066873), showing a difference in GES type. Both plasmids had mobilization elements (*mob* genes).

**FIG 1:**
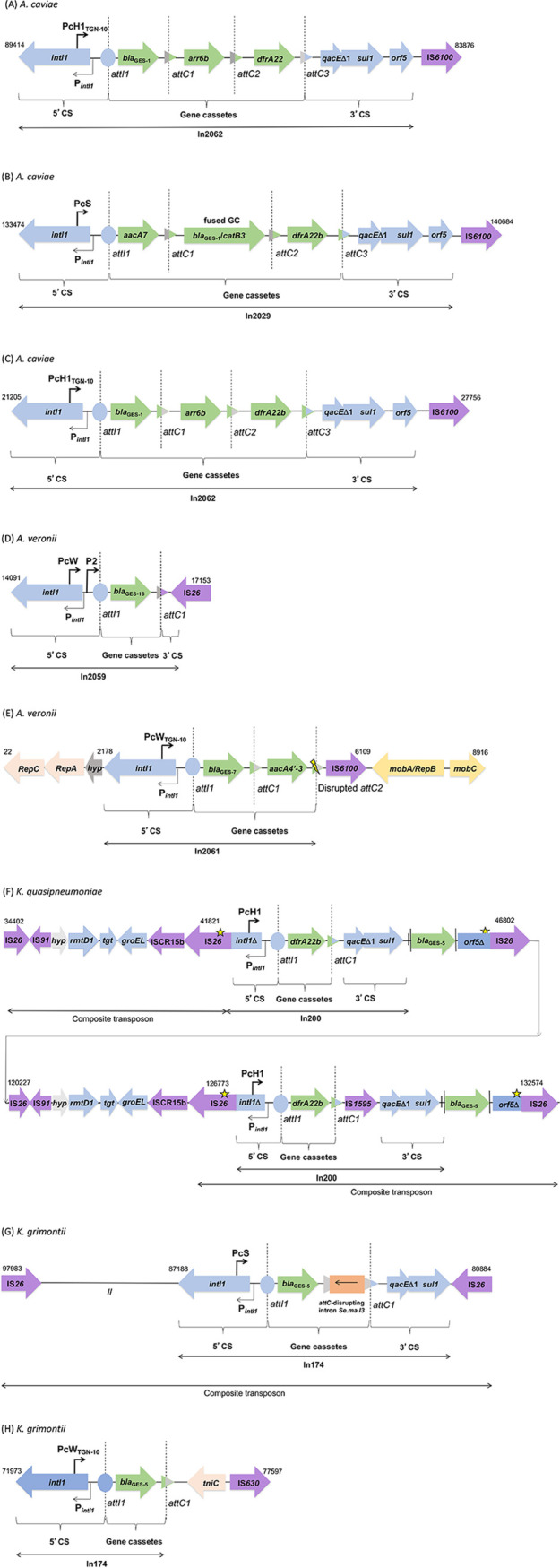
Schematic representation of regions enclosing class 1 integrons detected among the bacterial population analyzed in the present survey. (A) A. caviae (Aero19) In2062; (B) A. caviae (Aero21) In2029; (C) A. caviae (Aero52) In2062; (D) A. veronii (Aero22) In2059. The 3′ CS in In2059 is truncated by IS*26*. (E) A. veronii (Aero28) In2061. The yellow ray means the *attC* is disrupted, and therefore, the gene cassette cannot be mobilized anymore. The 3′ CS is truncated by IS*6100*. (F) K. quasipneumoniae (KPN47) In200. A star means premature *intI1* STOP codon and frameshift in *orf5*. (G) K. grimontii (p2KOX60), In174; (H) K. grimontii (p3KOX60), In174. Blue, conserved 5′ CS and 3′ CS; green, gene cassettes; purple, insertion sequences; //, 10-kb gap with different genes.

A conjugative plasmid (p1KPN47) from K. quasipneumoniae (IncM1) showed the BLASTn analysis similarity (86% coverage and 99.99% identity) with a clinical K. pneumoniae isolate (GenBank accession no. KT935445) (21, 22). Similar transfer regions and replication initiation and partitioning genes were found. Both plasmids were identified in isolates from Brazil and characterized as coproducers of carbapenemases and 16S-RMTase, with multiple copies of IS*26* and *rmtD1*-contanining regions duplicated in tandem (Fig. 1F). Among chromosome-related resistant genes, an *rmtD* copy was also recognized in K. quasipneumoniae (Table 2). Nucleotide insertions in the loop three regions of OmpK36 and OmpK37 were observed by DNA sequencing, and this type of mutation increases carbapenem MICs.

**FIG 2:**
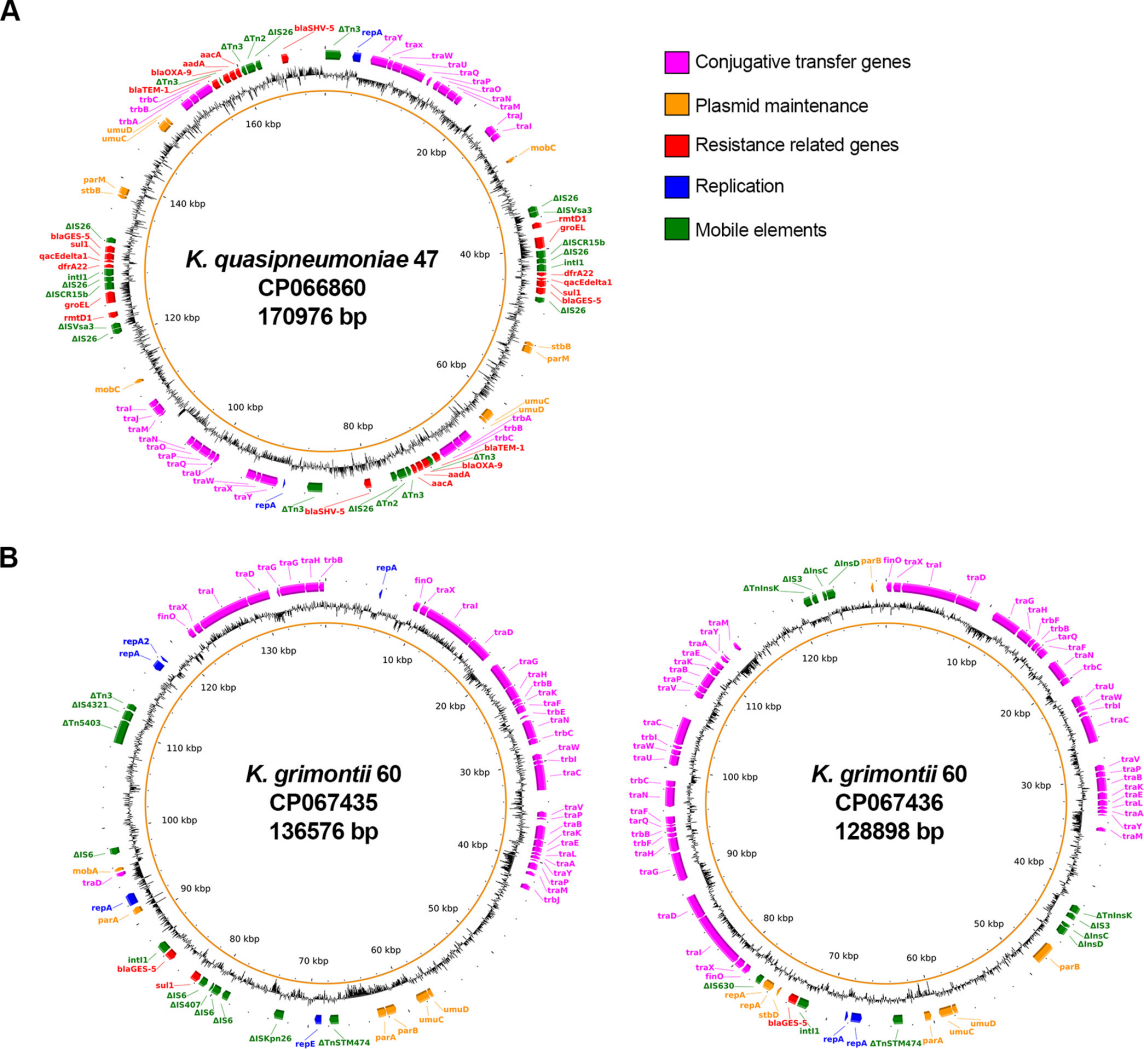
Map of the plasmids carrying *bla*_GES-5_ from KPN47 and KOX60 recovery at CHC/UFPR effluents. (A) The representative genes of the K. quasipneumoniae from the CP066860 plasmid are shown in colored boxes. (B) The representative genes of the K. grimontii from the CP067435 plasmid and CP067436 plasmid are shown in colored boxes.

K. grimontii carried two conjugative distinct plasmids carrying *bla*_GES-5_ (p2KOX60 and p3KOX60) (Fig. 2). The p2KOX60 harbored multiple replicons (FII-FIA) and displayed similarity (80% coverage and 99.99% identity) with a plasmid from wastewater K. grimontii strain (GenBank accession no. CP055312). The p3KOX60 carried an FII replicon and exhibited similarity (74% coverage and 99.14% identity) with a plasmid from freshwater Klebsiella michiganensis strain (GenBank accession no. CP058121). The main difference between these plasmids was the presence of integrons carrying *bla*_GES_.

By PFGE-S1 experiment and hybrid assembly, we found a unique plasmid in Aero19 (110 kb) and Aero21 (244 kb), 2 plasmids in Aero28 (9 and 59 kb), 3 plasmids in Aero22 (5.8, 23.9, and 80.5 kb) and Aero52 (9.7, 24.1, and 111.6 kb), 8 plasmids in KOX60 (ranging from 48 to 179.9 kb), and 10 plasmids in KPN47 (ranging from 10.2 to 170.9 kb).

### Novel class 1 integrons carrying *bla*_GES_ in *Aeromonas* spp.

The hybrid assembly analysis revealed that *bla*_GES_ from *Aeromonas* spp. was located in novel functional class 1 integrons (Fig. 1). Integrons with new and completely sequenced gene cassette arrays were considered for the attribution of new In numbers (23).

The In2062 (Fig. 1A and C) from A. caviae (Aero19 and Aero52) contained a *bla*_GES-1_-*arr6b*-*dfrA22* gene cassette array. Both integrons harbored the PcH1_TGN-10_ promoter variant (3, 23). In2029 (Fig. 1B) from A. caviae (Aero21) presented three gene cassettes, *aacA7*-*bla*_GES-1_ and *catB3*-*dfrA22*. The *bla*_GES-1_ and *catB3* cassettes were fused. This integron contained the strongest PcS variant. Both integrons carried a 5′ conserved segment (5′ CS) and 3′ conserved segment (3′ CS), followed by an IS*6100*.

In2059 (Fig. 1D) from A. veronii (Aero22) consisted of a weak variant (PcW) combined with a second promoter (P2), and the variable region comprised one gene cassette, *bla*_GES-16_, and a 3′ CS truncated by IS*26*. In2061 (Fig. 1E) was identified in A. veronii (Aero28) containing *bla*_GES-7_-*aacA4*′-*3* gene cassettes and presented the strong PcW_TGN-10_ variant (3, 23). The *attC* of *aacA4′-3* is disrupted, and, therefore, the gene cassette cannot be mobilized anymore. The 3′ CS region was absent, and IS*6100* was adjacent to the gene cassette.

### Genetic plasticity in known class 1 integrons carrying *bla*_GES-5_ in Klebsiella spp.

The plasmid of K. quasipneumoniae (KPN47) displayed two copies of the In200 class 1 integron (GenBank accession no. AJ968952) and presented *dfrA22b* as gene cassettes, both with a PcH1promoter variant (3, 23). The *bla*_GES-5_ gene, usually located as an integron gene cassette, was found between the 3′ CS and *orf5*Δ but was not associated with any *attC* and, therefore, not embedded as a mobilizable integron gene cassette. We observed the presence of an IS*1595* truncating the gene cassette, which may have caused the displacement of *bla*_GES-5_. The first 113 bp of the 5′ CS were deleted due to IS*26* insertion (IS*26*/Δ5′ CS); the 3′ CS included a truncated *orf5*Δ and an IS*26* (Fig. 1F).

K. grimontii (KOX60) exhibited two previously reported class 1 integrons, usually referred to as In174 (GenBank accession no. EU266532), containing one gene cassette (*bla*_GES-5_) in two different plasmids. In174 from p2KOX60 presented a strong promoter PcS and a novel group IIc intron that displayed 77% nucleotide identity with *Se.ma.I3* (GenBank accession no. AY884051) disrupting *attC* upstream of the 3′ CS region. This integron was bounded by two copies of IS*26*, forming a composite transposon (Fig. 1G). The second integron from p3KOX60 presented the strong PcW_TGN-10_ promoter variant and terminated in a *tniC* instead of a 3′ CS (Fig. 1H).

### Phylogenetic characteristic.

Multilocus sequence typing (MLST) based on the genomic data assigned six isolates to different STs, A. caviae (Aero19 and Aero52) ST884, A. caviae (Aero21) ST885, A. veronii (Aero22) ST886 (https://pubmlst.org/organisms/aeromonas-spp), K. quasipneumoniae (KPN47) ST5527 (http://bigsdb.pasteur.fr/), and K. grimontii (KOX6060) ST350 (https://pubmlst.org/organisms/klebsiella-oxytoca). The isolate A. veronii (Aero28) belongs to ST257 identified in China in 2013 (https://pubmlst.org/organisms/aeromonas-spp) (Table 2). KPN47 and KOX60 isolates, initially identified as K. pneumoniae and Klebsiella oxytoca by matrix-assisted laser desorption ionization–time of flight mass spectrometry (MALDI-TOF MS) (18), were redefined based on whole-genome sequencing as K. quasipneumoniae and K. grimontii, respectively.

## DISCUSSION

This study reports different GES-type enzymes from hospital effluents. The epidemiology of GES producers is not completely known, and its prevalence or transmission seems to be underestimated. However, there are some reports of environmental bacteria such as *Aeromonas* spp. (19, 24, 25). Our findings showed great potential of the environment in promoting genetic variability, a fact observed by the presence of novel integrons and the plasticity among the plasmids found. These scenarios are more common in natural environments than clinical settings, suggesting a general role in bacterial adaptation (26).

Antimicrobial resistance genomic analysis revealed that Aeromonas veronii isolates possess chromosomally encoded genes, including *bla*_cphA3_. As a resultant, this led to increased resistance to carbapenems. The Aero28 isolate presented also showed resistance to the novel carbapenem-β-lactamase inhibitor combinations, suggesting that, unlike in Aero22, an induction of the *cphA* gene may have occurred (27). The absence of this gene led to the reduction of MIC to carbapenems observed in GES-7 and GES-16 transformants. In addition, the MIC of ceftazidime in the GES-16 transformant dramatically declined. Some studies showed that the substitution of Gly170Ser in GES-carbapenemase reduces the hydrolytic efficiency against ceftazidime (28–31).

In Brazil, the presence of *bla*_GES-16_ was first reported in two carbapenem-resistant Serratia marcescens clinical isolates in Rio de Janeiro and did not show any relationship with the integron found in our study (28). We identified a novel mobilizable IncP6 plasmid carrying a novel integron that, compared to pKRA-GES-5 (20), had additional acquired resistance genes like *tetC*, *mphE*, *msrE*, and *bla*_TLA-1_ and a large number of mobile elements such as ISs, composite transposon, and different integrons. This plasmid group was reported in several species from clinical and wastewater sources, suggesting that they have a vast range of hosts and are associated with bacteria that can persist in the environment for long periods (32).

Genomic analysis of Aero28 showed that the integron (In2061) carrying *bla*_GES-7_ belongs to a novel IncQ1 plasmid, which is capable of replication in a very broad range of hosts and is readily mobilizable (33). The comparative BLAST analysis showed that this plasmid was homologous to another plasmid from a K. variicola clinical isolate harboring *bla*_GES-5_, deposited by our group in the GenBank database. These plasmids did not show significant homology with the other published IncQ-type plasmids carrying *bla*_GES_ that are inserted in different mobile elements, two from clinical origin (34, 35) and one from river water (36). Therefore, these findings highlight the spread of the IncQ1 plasmid between different species and environments.

The two isolates of Klebsiella spp. showed resistance to carbapenems and, as observed in GES-16, the β-lactam and aminoglycoside resistance decreased in the GES-5 transformant. Other mechanisms, such as porin loss in KOX60 or modifications in porins such as OmpK36 and OmpK37 in KPN47, are critical for expression of high-level resistance to carbapenems (17).

Members of the genus Klebsiella are known to have stronger associations between ISs and ARGs. Persistent exposure to antibiotics has likely enhanced this association of ISs with ARGs in their genomes (37). In the K. quasipneumoniae isolate (KPN47), a high frequency of IS*26* was noted. Furthermore, the ability of IS*26* transposase (Tnp26) to function in replicative mode formed two identical large regions of DNA sequences carrying *bla*_GES-5_ within class 1 integrons and arrays of important ARGs (2). This plasmid contains a wide range of transposable elements, an important evolutionary feature allowing for frequent genetic transposition leading to a plasmid fusion and, presumably, a better adaptation of the plasmid to the bacterial host (38). A similar situation involving *bla*_GES-5_ was recently reported in C. freundii isolated from a hospital wastewater treatment plant in Taiwan (39), drawing attention to hospital effluents.

Another interesting insight in K. quasipneumoniae isolate refers to identifying 16S rRNA-methyltransferase (*rmtD1*) in both plasmid and chromosome, showing a high resistance level against all aminoglycosides. It is noteworthy that most genes encoding 16S RMTases are typically located on plasmids that also encode ESBLs and carbapenemases determinants (40). Comparing the genetic environment of *rmtD1* with pKp64/11, we observed, in both plasmids, an *rmtD1-*containing region flanked by IS*26*, forming composite transposons. IS*26* may have played a role in the mobilization of *rmtD1* between different species (22, 41). Consequently, the duplication of IS*26*-flanked structures in a tandem array formation has been observed in the presence of selective pressure from the corresponding antimicrobial agents (22).

The duplicity of *bla*_GES-5_ was also observed in K. grimontii (KOX60), presented as gene cassettes in class 1 integrons in two distinct plasmids, as recently reported in a P. aeruginosa clinical isolate harboring two plasmids carrying *bla*_IMP_ (42). One integron (In174, p2KOX60) was located in a composite transposon (IS*26*), and the second (In174, p3KOX60), despite having the same gene cassette array, presented a different promoter variant and was not flanked by IS*26*, showing that these integrons are unrelated (39). Carbapenem-resistant K. grimontii (KPC-2) has been reported once, recovered from clinical samples in China (43), but K. grimontii carrying *bla*_GES-5_ recovered from hospital effluent in the first report.

By analyzing the complete sequence of the two IncF plasmids, we note the presence of multireplication proteins in p2KOX60 (*repA* and *repE*), featuring a replicon-type IncFII-FIA, whereas p3KOX60 had only *repA*. The presence of multiple replicons can allow those that are not driving replication to diverge, potentially changing incompatibility (44). These replication proteins, partitioning genes, and *tra* genes were responsible for the similarity of our plasmids to others recovered from the water environment in the United Kingdom. Our plasmids differ mainly by the acquisition of carbapenem resistance genes.

Although several Pc variants with different strengths were found in our isolates, including a Pc promoter combined with a second P2 promoter (Aero22), we were unable to observe a significant change in the MICs of the transconjugants. Notably, in the context of greater antibiotic selective pressure, the need to express gene cassettes more efficiently may lead to the selection of more efficient Pc sequences (3, 45). Further experimental studies covering the relative strengths of these Pc variant promoters should be performed.

Among all isolates, we achieved a transconjugant only for A. caviae (Aero21). Based on sequence analysis, p1Aero21 exhibits machinery needed for conjugation, all *tra* genes, and the type IV secretion system (T4SS). Genes transfer by conjugation is known to contribute to the genetic dynamics of bacterial populations living in a variety of environments (46). In the K. grimontii plasmid, we observed the presence of fertility inhibition protein (FinO) that may have decreased conjugation efficiency (2, 47). Further, in the K. quasipneumoniae plasmid, despite the presence of a *tra* region that included genes essential for F transfer, the conjugation was unsuccessful.

In summary, our study reports different *bla*_GES_ in novel plasmids within novel class 1 integrons and different STs of *Aeromonas* spp. and Klebsiella spp. recovered from hospital effluents, reflecting what could be found within the patient’s gut. We share an in-depth exploration of understanding the molecular evolutionary mechanisms of *bla*_GES_ mobilome, along with their potential dynamic transmission and plasmid plasticity.

The presence of novel mobilizable or conjugative plasmids under different contexts, including an impressive mesh of interactions with transposable elements, resulting in plasmid fusion and acquisition of multireplicons, can translate into a problem of large proportion. This is because wastewater treatment plants cannot eliminate these MGEs and antibiotic-resistant bacteria (ARB) that can persist in river water and thus constitute a threat of dissemination in the environment.

## MATERIALS AND METHODS

### Study settings and ethics statement.

The Institutional Ethics Review Board of the Complexo Hospital de Clínicas, Universidade Federal do Paraná (CHC/UFPR), approved this study under the reference number CAAE 11087012.0.0000.0096.

### Characterization of bacterial isolates and antimicrobial resistance.

We selected a group of seven GES type-producing isolates from hospital effluents, as identified in previous studies (18, 19). Wastewater samples were collected at the CHC/UFPR, a 640-bed academic care hospital, and Hospital Pequeno Principe (HPP), a 390-bed pediatric academic care hospital. The hospitals are located in Curitiba, Paraná, southern Brazil. These isolates were identified using the Vitek 2 compact system (bioMérieux, Marcy-l’Étoile, France) and mass spectrometry (matrix-assisted laser desorption ionization–time of flight mass spectrometry [MALDI-TOF MS]) using microflex LT Biotyper 3.0 (Bruker Daltonics, Bremen, Germany) and Vitek MS (bioMérieux) instruments. All isolates were stored in 15% glycerol-containing tryptic soy broth and frozen at −80°C until further use.

Antimicrobial susceptibility testing was performed by agar or broth dilution as recommended by CLSI (48, 49). The tests were interpreted according to CLSI standards (49, 50). Double-disk synergy was assessed to detect ESBL (51) and class A and B carbapenemases (52).

### Plasmid profile and translocation of *bla*_GES_.

Total plasmid DNA extraction of Klebsiella species and *Aeromonas* species isolates harboring *bla*_GES_ was performed using the GenElute plasmid midiprep kit (Sigma-Aldrich, St. Louis, MO, USA) according to the manufacturer’s instructions. To define the size and number of plasmids in each isolate, S1 nuclease pulsed-field gel electrophoresis (PFGE-S1) was performed as described by Kaufmann (53). Conjugation experiments were performed using *Aeromonas* and Klebsiella isolates as donors and azide-resistant E. coli J53 as receptor strain. Transconjugants were selected on MacConkey agar containing 150 mg/L sodium azide and 50 mg/L ampicilin. Additionally, DNA plasmids obtained from the extraction by NucleoBond Xtra Plus midikit (Macherey-Nagel, Duren, Germany) were transformed by electroporation into E. coli TOP10. Electroporation conditions were 100 Ω, 13 kV/cm, and 25 μF (54), and transformants were selected with ceftazidime (0.5 mg/L).

### Genome sequencing, assembly, and annotation.

Genomic DNA was extracted using the GenElute bacterial genomic DNA kit (Sigma-Aldrich) and quantified using the Qubit double-stranded DNA (dsDNA) high-sensitivity (HS) assay kit (Thermo Fisher Scientific Inc., Waltham, USA). Illumina sequencing libraries with an average insert size of 600-bp fragments were generated using an Illumina Nextera XT DNA library kit (Illumina Inc., San Diego, CA, USA). Libraries were quantified, and their quality was verified using a Bioanalyzer 2100 system (Agilent Technologies, Santa Clara, CA, USA). Whole-genome sequencing of paired-end libraries (PE; 2 × 250 bp) was performed using the Illumina MiSeq platform (Illumina Inc.). In addition, long-read WGS was performed using a MinION sequencer (Nanopore, Oxford, UK). MinION sample preparation was carried out using the rapid barcoding kit SQK-RBK-004 and the flow cell priming kit EXP-FLP002 (Oxford Nanopore), following the manufacturer’s instructions.

Raw read quality was checked using FastQC version 0.11.15 (https://www.bioinformatics.babraham.ac.uk/projects/fastqc/), and quality-based trimming and filtering were performed using Trimmomatic version 0.35 (55). SPAdes version 3.12.0 (56) was used to combine MinIon and Illumina data to produce a hybrid assembly of chromosomes and plasmids. The number of contigs was more contiguous than the assembly using Illumina data alone, with SPAdes producing a single chromosomal contig. Completeness and taxonomic classification of the assemblies were verified using the DFAST tool (https://dfast.nig.ac.jp/dqc/submit/; accessed in September 2020). Chromosomal and plasmid sequences were annotated using Prokka version 1.12 (57). Rapid Annotation using Subsystem Technology (RAST) version 1.073 (58) and Artemis version 18.0.0 (59) were applied to predict coding sequences (CDSs). We searched all the plasmids of the GenBank database (accessed in May 2022) using BLASTn (60). Comparative plasmid maps of *bla*_GES-5_ were generated from the assembled contigs using BLAST Ring Image Generator (BRIG) version 0.95 (61).

### Profiling of mobile genetic elements and antimicrobial resistance genes.

Chromosomal and plasmid resistome were predicted using the Comprehensive Antibiotic Resistance Database (CARD) version 3.1.2 (https://card.mcmaster.ca/, accessed January 2020) and ResFinder database version 4.1 (https://cge.cbs.dtu.dk/services/ResFinder/, accessed December 2020).

Plasmid replicon types in the assemblies were determined using the PlasmidFinder tool 2.0.1 (https://cge.cbs.dtu.dk/services/PlasmidFinder/, accessed December 2020). Moreover, the allele types of IncF plasmids were assigned using IncF replicon typing pMLST, available at the Center for Genomic Epidemiology (https://cge.cbs.dtu.dk/services/pMLST/, accessed December 2020). Chromosomal and plasmid transposons were identified using Mobile Element Finder 1.0.3 (https://cge.cbs.dtu.dk/services/MobileElementFinder/, accessed December 2020) and ISfinder (https://isfinder.biotoul.fr/index.php, accessed January 2021). Plasmid integrons, integrases, and gene cassettes were predicted using the INTEGRALL database version 1.2 (http://integrall.bio.ua.pt/, accessed June 2021).

### Molecular typing.

Sequences of housekeeping genes of unknown STs were curated at the K. pneumoniae MLST database at the Pasteur Institute (http://bigsdb.pasteur.fr/), the K. grimontii MLST website (https://pubmlst.org/organisms/klebsiella-oxytoca), and the *Aeromonas* spp. MLST website (https://pubmlst.org/organisms/aeromonas-spp).

### Data availability.

Genomic assemblies of chromosomes and plasmids of the seven isolates were submitted to GenBank under the accession numbers CP068232 and CP068233 (Aeromonas caviae 19), CP068231 (Aeromonas caviae 21), JAEMTZ000000000 (Aeromonas veronii 22), JAEMUA000000000 (Aeromonas veronii 28), CP066813 to CP066816 (Aeromonas caviae 52), CP066859 to CP066869 (Klebsiella quasipneumoniae 47), and CP067433 to CP067441 (Klebsiella grimontii KOX 60).
